# Supplementary material for: Venomix: a simple bioinformatic pipeline for identifying and characterizing toxin gene candidates from transcriptomic data
Source: PeerJ. 2018 Jul 31;6:e5361. doi: 10.7717/peerj.5361 (PMC6074769; doi:10.7717/peerj.5361)
Supplement: Supplemental Information 3 [file peerj-06-5361-s003.gz › FinalOutput_E-6/Factor_V_activator_RVV-V_gamma_142/finaltree.pdf]

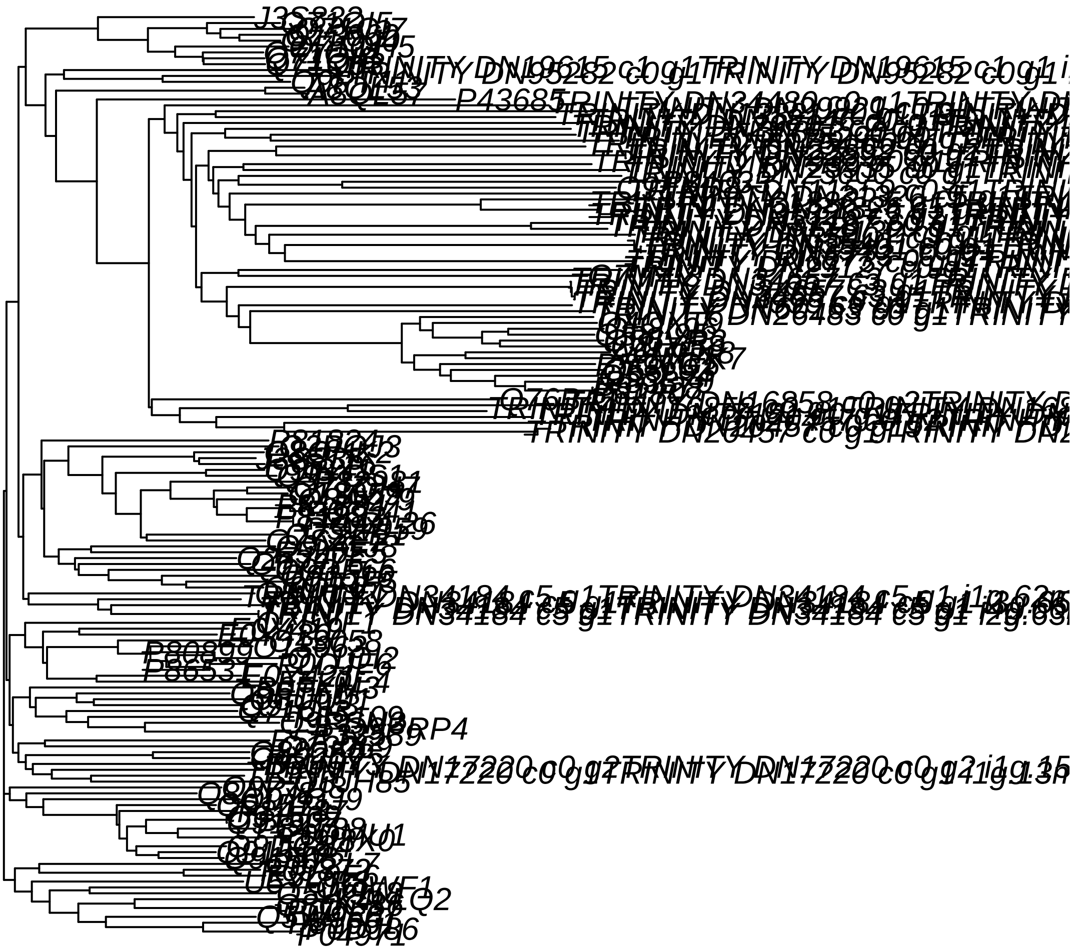
J3S832
P43685
TRINITY DN20483 c0 g1TRINITY
TRINITY DN20457 c0 g1TRINITY DN2
